# The hidden microbial ecosystem in the perennial ice from a Pyrenean ice cave

**DOI:** 10.3389/fmicb.2023.1110091

**Published:** 2023-01-26

**Authors:** Fátima Ruiz-Blas, Víctor Muñoz-Hisado, Eva Garcia-Lopez, Ana Moreno, Miguel Bartolomé, Maria Leunda, Emma Martinez-Alonso, Alberto Alcázar, Cristina Cid

**Affiliations:** ^1^Centro de Astrobiología (CAB), CSIC-INTA, Madrid, Spain; ^2^Section Geomicrobiology, GFZ German Research Centre for Geosciences, Potsdam, Germany; ^3^Departamento de Procesos Geoambientales y Cambio Global, Instituto Pirenaico de Ecología - CSIC, Zaragoza, Spain; ^4^Institut für Geologie und Mineralogie, Universität zu Köln, Köln, Germany; ^5^Institute of Plant Sciences and Oeschger Centre for Climate Change Research, University of Bern, Bern, Switzerland; ^6^Swiss Federal Research Institute for Forest, Snow and Landscape Research WSL, Zurich, Switzerland; ^7^Department of Plant Biology and Ecology, University of the Basque Country, Leioa, Spain; ^8^Department of Investigation, Instituto Ramón y Cajal de Investigación Sanitaria, Hospital Ramón y Cajal, Madrid, Spain

**Keywords:** ice cave, Pyrenees, global warming, microbial community profiling, next-generation sequencing, environmental variables, proteomics

## Abstract

Over the last years, perennial ice deposits located within caves have awakened interest as places to study microbial communities since they represent unique cryospheric archives of climate change. Since the beginning of the twentieth century, the temperature has gradually increased, and it is estimated that by the end of this century the increase in average temperature could be around 4.0°C. In this context of global warming the ice deposits of the Pyrenean caves are undergoing a significant regression. Among this type of caves, that on the Cotiella Massif in the Southern Pyrenees is one of the southernmost studied in Europe. These types of caves house microbial communities which have so far been barely explored, and therefore their study is necessary. In this work, the microbial communities of the Pyrenean ice cave A294 were identified using metabarcoding techniques. In addition, research work was carried out to analyze how the age and composition of the ice affect the composition of the bacterial and microeukaryotic populations. Finally, the *in vivo* effect of climate change on the cellular machinery that allow microorganisms to live with increasing temperatures has been studied using proteomic techniques.

## Introduction

In recent years much research has been done on the microbiology of the cryosphere (Boetius et al., 2015), especially ice sheets of Arctic and Antarctica, polar marine ice shelves, mountain glaciers, ice lakes, deep seas, and subglacial lakes (Garcia-Lopez and Cid, 2017; Garcia-Lopez et al., 2019). Nevertheless, perennial ice caves have been studied to a much lesser extent. Ice caves are defined as rock cavities hosting perennial ice that results from the diagenesis of snow and/or the freezing of infiltrating water through fissures (Perşoiu and Lauritzen, 2018). These environments represent a small portion of the total cryosphere (Kern and Perşoiu, 2013), yet they are an important source of paleoclimate and paleoenvironmental information (Kern et al., 2009; Stoffel et al., 2009; Feurdean et al., 2011; Spötl et al., 2013; Perşoiu et al., 2017; Sancho et al., 2018; Leunda et al., 2019; Racine et al., 2022) that is under imminent risk of disappearance (Kern and Perşoiu, 2013; Perşoiu et al., 2021; Wind et al., 2022). Over the last years, ice caves have awakened interest as places to study both modern and fossil ice microbiota communities (Iţcuş et al., 2016; Brad et al., 2018; Iţcuş et al., 2018; Mondini et al., 2019; Paun et al., 2019, 2021; Mulec et al., 2021). Still, the bacterial diversity of ice caves has been barely explored. Examples of researched caves include European limestone ice caves (Margesin et al., 2004; Hillebrand-Voiculescu et al., 2013, 2015; Iţcuş et al., 2018), icy volcanic environments in Oregon (Popa et al., 2012) and Hawaii (Teehera et al., 2018) and those near Mt. Erebus (Connell and Staudigel, 2013; Tebo et al., 2015). The study of these different microbial communities in various geological locations is relevant to know which adaptation mechanisms depend on the increase in temperature and which depend on the location and chemical composition of the samples. These microbial communities live in a very stable environment. They are protected from external agents such as light, wind, or precipitation. Living confined to ice, they are not affected by water currents, and they are rich in nutrients from soil dust and animal waste. An increase in temperature in a very stable environment like a cave can modify the microbial population composition and their mechanisms of adaptation, metabolism, and biogeochemistry (García-Descalzo et al., 2022). Their molecular machinery may have been modified to adapt to temperature rise and to the changes in the chemical composition of the ice (Garcia-Lopez et al., 2016; Garcia-Lopez and Cid, 2017).

The existence of ice caves in northern Spain has been well known since the pioneering reconnaissance work in the Monte Perdido Massif (Central Pyrenees) in the mid-twentieth century (Casteret, 1953). Since the beginning of the twentieth century, the temperature has suffered a gradual increase of 1.3°C in the Pyrenean mountain range (OPCC-CTP, 2018), and the ice deposits of the Pyrenean caves are undergoing a significant regression (Belmonte-Ribas et al., 2014). The total glacierized area in the Pyrenees has shrunk by 23.2%, and thickness has decreased on average by 6.3 m between 2011 and 2020 (Vidaller et al., 2021). According to climate model estimates, the annual maximum temperature in the Pyrenees by 2050 will increase by 1–4°C compared to the 1986–2005 reference period in the Representative Concentration Pathway (RCP) 8.5 scenario. This RCP scenario is one of the possible greenhouse gas concentration trajectories, and depends on the volume of greenhouse gases emitted in the upcoming years (van Vuuren et al., 2011; Amblar-Francés et al., 2020; Bilbao Barrenetxea and Faria, 2022).

In this research work, microorganisms from the A294 cave ice were identified and cultivated at two different temperatures (0 and 4°C), emulating the warming scenario to which these populations could be exposed to. Their metaproteome was studied to understand how they adapt their molecular machinery to increasing temperatures.

This work has important implications on the microbiology of the cryosphere and its alteration by climate change, and tries to explain interesting questions such as (i) which microorganisms live in perennial ice caves, (ii) how climate change affects them, (iii) how their molecular machinery adapts to increases in temperature, and (iv) how their metabolism varies in response to temperature rises.

## Materials and methods

### Study site: The A294 ice cave

**FIGURE 1:**
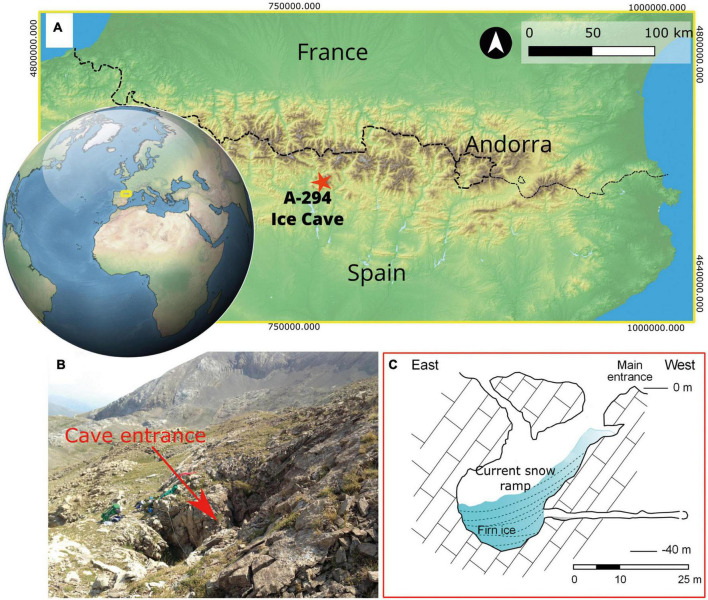
Geological setting of samples. **(A)** Location of the A294 ice cave in the Central Pyrenees. **(B)** Photograph showing the main entrance of the cave. **(C)** Profile view of the A294 ice cave showing the snow ramp and the position of the ice body, modified from Belmonte-Ribas et al. (2014).

A294 ice cave (UTM coord. 31T 0281171 4710349, 2238 m a.s.l) is located within the Armeña cirque in the Cotiella massif (Central Pyrenees, NE of Iberia; Figure 1A). A294 is a small sag-type cave (∼33 m deep) with two vertical entrances, which allows the snow inlet (Figures 1B, C). The small entrance is usually sealed during the winter season, while the main one (∼30 m^2^; Figure 1B) remains open all year long. A snow ramp connects the main entrance with the top part of the fossil ice inside the cave (Figure 1C). According to Sancho et al. (2018), the temperature inside the cave ranges between −0.77°C in winter (November–May) and 0.26°C in summer (June–October). This ice constitutes the world’s oldest (6,100 ± 107 year cal. BP) known deposit of firn in a cave (Sancho et al., 2012, 2018; Leunda et al., 2019). The fossil ice body (∼9 m in 2011) spanned between 6,100 and 1,880 years. The ice body has suffered an important retreat over the last years, as shown by ice measurements and picture comparison (Belmonte-Ribas et al., 2014; Leunda et al., 2019).

### Ice drilling, sampling, and radiocarbon dating

**FIGURE 2:**
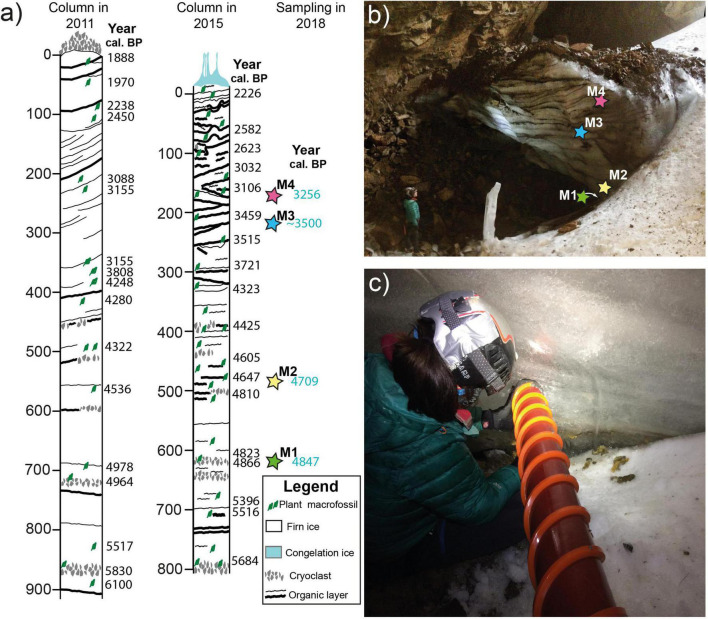
Stratigraphy and ice sampling. **(a)** Stratigraphy and chronology of the ice deposit in 2011 (Sancho et al., 2018) and 2015 (Leunda et al., 2019) together with the position of the samples taken in 2018 for the present study. **(b)** View of the ice body in 2018 and the approximate position of the samples taken. **(c)** Ice sampling using the ice auger. M1, M2, M3, and M4, names of the samples; Cal. BP, calibrated years before the present.

Four ice core samples (M1, M2, M3, and M4) were taken from the ice body in July 2018, using a 9 cm diameter-1-m-long Mark II ice auger (Kovacs) to perform microbiological analyses (Figure 2). The retreat of the ice body did not allow correlating these new four samples with the previously dated ice stratigraphy published in Sancho et al. (2018) and Leunda et al. (2019). Thus, in order to know the age of M1, M2, M3, and M4 additional ice samples at the same depths for accelerator mass spectrometry (AMS) radiocarbon analyses were extracted (except for sample M3, where the same layer could be identified). Terrestrial plant macrofossils were selected for AMS radiocarbon dating, and the analyses were performed at the AMS Direct laboratory facilities (Seattle, USA). The ^14^C dates were converted to calibrated ages (cal. BP) in R (version 3.6.0; R Core Team, 2020) using the package *clam* (Blaauw, 2010) with the IntCal20 calibration curve (Reimer et al., 2020).

A summary of the overall experimental strategy is represented in Supplementary Figure 1. Ice samples were immediately wrapped in sterile plastic bags as previously reported (Moreno et al., 2021), and transported at −20°C from the field to the laboratory at the Centro de Astrobiología (Madrid, Spain). Then, ice samples were decontaminated following previously described procedures (Martinez-Alonso et al., 2019). A section of ice core was removed from −20°C and soaked in ice-cold 95% ethanol for 1 min, followed by extensive rinsing with 0.22 μm-filtered MilliQ water. The exterior 3-cm shell of ice samples (corresponding to 30% of total ice volume) was ablated. Previous work had shown that these procedures are effective in removing surface contamination (Rogers et al., 2004; Christner et al., 2005). Only the inner ice of each core was thawed at 4°C inside a sterile plastic bag and used in the analyses. A laboratory contamination control was performed with a 1 l of MilliQ water that was frozen, thawed, filtered, and subjected to all analytical procedures including DNA extraction, PCR, sequencing, and culturing. All procedures were performed by using bleach-sterilized work areas, a UV-irradiated laminar flow hood, ethanol-sterilized tools, and sterilized gloves.

Each ice core was cut into three parts obtaining 3 sampling replicates. Thus, a total of 12 samples: 4 samples (M1, M2, M3, and M4) with 3 replicates (named a, b, c) were analyzed. Protocols for aseptic sampling and the tracing of potential contaminations were followed. The meltwater samples were either individually used for cultures or filtered through filters with pores of 0.22 μm attached to a vacuum pump in a flow hood, previously sterilized with ethanol. Both filters and meltwater were used for DNA extraction and chemical analysis.

### Chemical analysis of ice samples

Assays for putative nutrients such as NH_4_^+^, NO_2_^–^, NO_3_^–^, SO_4_^2–^, soluble reactive phosphorus (SRP), and dissolved organic carbon (DOC) from each filtered meltwater sample were performed by ion chromatography in an 861 Advance Compact IC system (Metrohm AG, Herisau, Switzerland). Ions in ice samples were identified and quantified with internal and external standards prepared from Certified Standard Solutions (TraceCERT^®^) (Merck). Chromatograms were analyzed with the Metrohm IC Net 2.3 SR4 software (Garcia-Lopez et al., 2022). Detection limits for these constituents ranged from 0.1 to 2.0 μM. Concentrations of ions were analyzed by inductively coupled plasma-mass spectrometry (ICP-MS) on a Perkin Elmer ELAN9000 ICP-MS quadrupole spectrometer (Martinez-Alonso et al., 2019). Values > 0.999 ppb were considered for the statistical test.

### Extraction, quantification, and sequencing of DNA

The DNA from each 0.22 μm pore filter was extracted and purified with a DNA Isolation PowerWater kit (MO BIO Laboratory, Inc.). Extraction procedures were identical for all samples. DNA concentration was determined using a NanoDrop 2000p. The diversity of uncultured microeukaryotes and bacteria was assessed by Illumina MiSeq 16S and 18S rRNA gene amplicon sequencing. The amplification and sequencing of the V3–V4 regions of the 16S rRNA gene (forward sequence CCTACGGGNGGCWGCAG; reverse sequence GACTACHVGGGTATCTAATC) were performed to identify bacteria. Microeukaryotes were identified by amplification and sequencing of the V4–V5 regions of the 18S rRNA gene (forward sequence GCCAGCAVCYGCGGTAAY; reverse sequence CCGTCAATTHCTTYAART).

### Metabarcoding data processing

Quality analyses of reads were performed using FastQ Screen software (version 2) (Wingett and Andrews, 2018). Contigs were trimmed to include only the overlapping regions using PANDAseq Assembler (Bartram et al., 2011). This software does also remove the sequence of the primers, discarding the pairs that do not have primer sequences. For the analysis, the QIIME2 (2022.8) software was used (Bolyen et al., 2019). The sequences of all samples were grouped to define the amplicon sequence variants (ASVs) with DADA2. The quality of the initial bases was high in the demux.qzv quality plots, and the value dropped off around position 280. Therefore, the settings used were –p-trim-left 0 \; –p-trunc-len 280 \. Sequences were aligned against the SILVA 138.1 database. The Blast method was used to assign taxonomy.

### Cultured-metaproteomics

This study combines cellular cultures and proteomic approaches, in an attempt to simulate the molecular adaptation of cave microorganisms to warming temperatures. During the summer and part of the fall season, the cave maintains temperatures close to 0°C. The estimated future temperature increase is of 4°C.

Both cultures (100 mL) were grown as in Martinez-Alonso et al. (2019). Nutrient formulations for T2, T3, T4, T6, T7, and T8 were prepared as concentrated stocks (20x), sterilized, and added to 100 mL of meltwater to get 1x final concentration as described in Bidle et al. (2007) (T2–T6), and Garcia-Descalzo et al. (2010) (T7–T8) (Supplementary Table 3). The cultures were carried out in Corning closed system bottles to avoid external contamination during the cultivation time. Each sample was incubated at 2 temperatures (0 ± 0.1 and 4 ± 0.1°C) in a cooled incubator (Memmert GmbH, Schwabach, Germany). Three replicates were cultured under each culture condition. Growth was monitored by optical density at 600 nm 20 days. All growth curves are represented in Supplementary Figure 2. A total of 48 cultures were obtained (4 ice samples in 6 culture media at 2 different temperatures). The culture medium in which the cells grew best was T5 (Supplementary Figure 2), and these cultures were used for the proteomics assays.

Cultures were centrifuged (10,000 × g, 15 min), rinsed in PBS and stored at −20°C. Afterward, proteins were extracted as explained in previous reports (Cid et al., 2010; Garcia-Descalzo et al., 2012). Proteins were analyzed using 2-dimensional electrophoresis (2-DE) by combining horizontal slab gel isoelectric focusing (IEF) with SDS-PAGE. Carrier ampholyte urea IEF was performed using pH 4–7 strips (11 cm). The spots resolved by 2-DE from the gels were stained with Coomassie Blue for peptide mass fingerprinting or MS/MS analysis and protein identification. Spectral data were analyzed to search them in the NCBI database using the Mascot search algorithm (Matrix Science, London, UK). Search parameters were: Enzyme: Trypsin; Fixed modifications: Carbamidomethyl (C); Variable modifications: Oxidation (M); Mass values: monoisotopic; Protein Mass: Unrestricted; Peptide Mass Tolerance: ± 80 ppm; Fragment Mass Tolerance: ± 0.3 Da; Max Missed Cleavages: 1; Instrument type: MALDI-TOF-TOF. The mass spectrometry proteomics data have been deposited in the ProteomeXchange Consortium^1^ via the PRIDE partner repository (Perez-Riverol et al., 2019) with the dataset identifier PXD029615.

### Statistical analysis

Statistical differences on the ion concentrations, number of ASVs and number of proteins among samples were tested under ANOVA test and Newman-Keuls Multiple Comparison post-test using GraphPad Prism version 7.0 (GraphPad Software, La Jolla California USA^2^). All data were expressed as media ± SD of three sampling replicates. The effects of the chemical composition of glacial ice, as well as the influence of the depth on the microbial community composition, were investigated by a combination of multivariate statistical analysis -Detrended Correspondence Analysis (DCA), Principal Components Analysis (PCA), and Canonical Correspondence Analysis (CCA)- developed with CANOCO 5 software (Microcomputer Power, Ithaca) (Ter Braak and Šmilauer, 2002). The parameters used in each analysis are summarized in Supplementary Table 4. Species data were not transformed, except in some specific analyses that are explained next. Monte Carlo tests with 500 permutations were run.

## Results and discussion

### General characteristics and chemical properties of the ice

The quantification of nitrogen, sulfur, and phosphorus ions is relevant, since these compounds are used by microorganisms as nutrients. The concentrations of NH_4_^+^, NO_2_^–^, NO_3_, SO_4_^2–^, SRP, and DOC in the ice samples were represented in Table 1. These analyzes showed that the concentrations of all these ions were significantly higher in the M3 sample (ANOVA test and Newman-Keuls Multiple Comparison post-test, *p* < 0.05**). These values were also higher in sample M4 (approximately double) than in the results for M1 and M2 (Table 1). This fact could be due to the distribution of organic plaques throughout the ice body, especially in the case of DOC, since samples M3 and M4 were taken in an area with more presence of organic matter. The samples were also analyzed by mass ICP to determine the presence of chemical elements that can be considered nutrients (for example C, Ca, or Fe) or potentially toxic elements (for example Cu or Zn). The results obtained were shown in Table 2.

**TABLE 1:** Chemical analysis of soluble nutrients in meltwater.

Sample	NH_4_^+^	NO_2_^–^	NO_3_^–^	SO_4_^2–^	SRP^a^	DOC^b^
M1	1.57 (0.12)	2.01 (0.23)	3.87 (2.01)	255.38 (20.17)	0.38 (0.08)	18.12 (1.38)
M2	0.98 (0.10)	1.28 (0.22)	2.00 (0.11)	197.35 (11.87)	0.29 (0.07)	15.28 (0.98)
M3	**6.28 (0.87)**	**6.98 (1.25)**	**12.65 (3.21)**	**998.33 (52.32)**	**1.58 (0.15)**	**108.33 (9.31)**
M4	2.57 (0.34)	3.28 (0.98)	5.21 (1.59)	551.32 (23.78)	0.97 (0.09)	36.28 (5.37)

Concentrations are expressed as μM (±SD) of three replicates. ^a^SRP, soluble reactive phosphorus; ^b^DOC, dissolved organic carbon. The highest nutrient values are marked in bold.

**TABLE 2:** Chemical analysis of ions in meltwater.

Sample	C	Na	Si	P	S	K	Ca	Mn	Fe	Cu	Zn	Mg
M1	8863.780	417.480	3.038	3.995	BD*	214.510	1834.200	4.746	2.098	1.456	**20**.**613**	29.441
M2	13366.930	**372.843**	2.820	**17**.**275**	1.438	**242**.**202**	2246.080	5.474	2.581	**3**.**615**	4.192	45.391
M3	12235.911	355.997	3.038	3.689	60.962	208.242	2143.820	**12**.**855**	1.207	2.949	6.490	39.559
M4	**90537**.**550**	132.201	**22**.**640**	3.221	**596**.**423**	88.346	**4044**.**790**	8.042	**3**.**311**	1.715	2.086	**74**.**857**

Concentrations are expressed in ppb (±SD) of three replicates. *BD, below detection. The highest nutrient values are marked in bold.

### Stratigraphy and chronology of the samples

The ice body is made of cross-stratified ice beds formed by the accumulation of the snow that entered the cave from the main entrance. The ice deposit includes detrital and organic−rich layers comprising cryoclastic rock fragments, fine detrital sediments, and plant remains (Figure 2A). The results of the age of the samples were in agreement with previous chronologies (Sancho et al., 2018; Leunda et al., 2019), ranging from ca. 4,850 to 3,260 (Table 3).

**TABLE 3:** Radiocarbon ages from terrestrial plant macrofossils from the A294 ice cave.

Sample	Radiocarbon age (^14^C year BP)	Calibrated age (2σ) (cal. year BP)	Median age (cal. year BP)
M1	4,286 ± 33	4,959–4,734	4,847
M2	4,179 ± 35	4,836–4,581	4,709
M3	−	−	3,500*
M4	3,036 ± 30	3,349–3,162	3,256

*From Leunda et al. (2019).

### Microbial community composition

**FIGURE 3:**
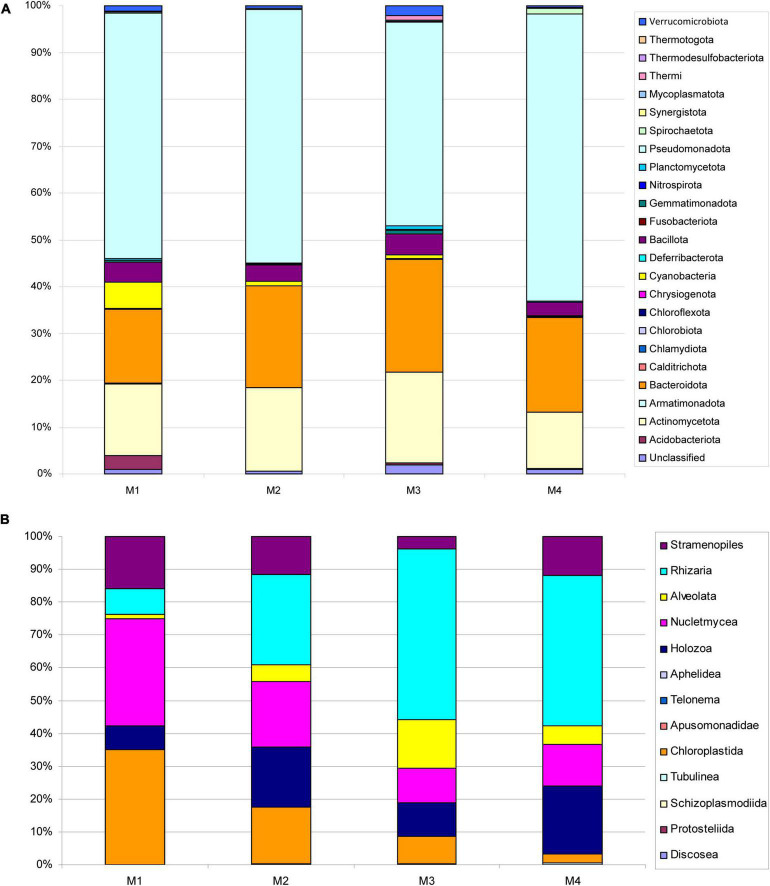
Microbial community distribution in the cave ice samples at the phylum level. Relative abundances of major taxa of bacteria and microeukaryotes based on **(A)** 16S rRNA and **(B)** 18S rRNA gene sequencing data, respectively.

In this study, a total of 183,724 bacterial ASVs were obtained which belonged to 1,495 species spanning 24 phyla. Only 1% of 16S rRNA gene amplicons corresponded to unidentified ASVs, indicating that the surveying effort covered almost the full extent of taxonomic diversity. *Pseudomonadota* (53%), *Bacteroidota* (21%), and *Actinomycetota* (16%) were the most abundant groups (Figure 3A). The most abundant bacterial genera were *Pedobacter*, *Rhodoferax*, *Cryobacterium*, *Oxalobacter*, and *Calothrix* (Supplementary Figure 3). This last genus was very abundant in M1. No significant differences were detected by ANOVA test between the numbers of ASVs among the four samples studied (*p* = 0.9943). The bacteria of the phylum *Pseudomonadota* were the most abundant in all samples. The *Bacteroidota* phylum was mainly identified in the most recent samples of the ice body (3,500–3,256 years old), while the *Actinomycetota* phylum was found principally in the innermost areas of the ice block (4,709–3,500 years old strata). *Cyanobacteria* were mostly identified in the first layer samples exposed to sunlight. In general, the results obtained in this study were consistent with previous work, in which these same phyla were found in samples from ice caves (Iţcuş et al., 2016, Iţcuş et al., 2018; Paun et al., 2021).

The distribution of microorganisms not only depended on the age of the sample but also on the organic composition of the ice, which provides a considerable source of nutrients. It could be observed that samples M3 and M4 were collected in areas where both the organic layers and the chemical composition of the ice were significantly different from the rest (Table 1 and Figure 2). In these samples the presence of organotrophic bacteria was much higher. For example, some species of *Caldithrix* and also several acetogens such as *Acetobacterium* and *Clostridium* (which can grow chemoorganotrophically by fermentation of sugars) were identified. Unexpectedly, thermophilic bacteria such as *Thermoanaerobacter* and *Thermodesulfovibrio* were found, especially in M4. These bacteria had already been identified in other ice samples from glacial environments (García-Lopez et al., 2021a,b).

Regarding microeukaryotes, very little diversity was observed. Although many ASVs (a total of 2,922,230) were obtained, only 142 species were identified, across 13 phyla. Most eukaryotic microorganisms belonged to *Rhizaria* (34%), *Nucletmycea* (19%), and *Chloroplastida* (15%) (Figure 3B). Most of the fungi (Nucletmycea) belonged to the phyla *Ascomycota* and *Basidiomycota*. The presence of fungi in ice caves had already been described in previous papers (Brad et al., 2018). Although the samples studied in those investigations were more modern (400–900 years old), the phyla found were similar.

In our samples, the distribution of microeukaryotes varied with ice age. Most of the microorganisms in the oldest samples (4,847–4,709 years old) were representatives of the phylum *Rhizaria*, while the most modern samples (3,500–3,256 years old) contained microeukaryotes of the phyla *Nucletmycea* and *Chloroplastida*. At the genus level, the most abundant in all the samples was the cercozoa *Heteromita*. This flagellate had already been identified as a very abundant genus in other ice samples (García-Descalzo et al., 2013; García-Lopez et al., 2021a). Its influence on the bacterial communities in which it excretes ammonium has been described (Murase et al., 2006). Furthermore, the ciliophore *Spirotrichea* was very abundant in the modern samples, but was not found in the ancient ones. The class *Spirotrichea* participates in a series of ecological and biogeochemical processes, including energy flux and nutrient remineralization (Santoferrara and McManus, 2017). So, they play important roles in the food web, where they consume bacteria, diatoms, and dinoflagellates; and are themselves ingested by small metazoans.

### Microbial community distribution

**FIGURE 4:**
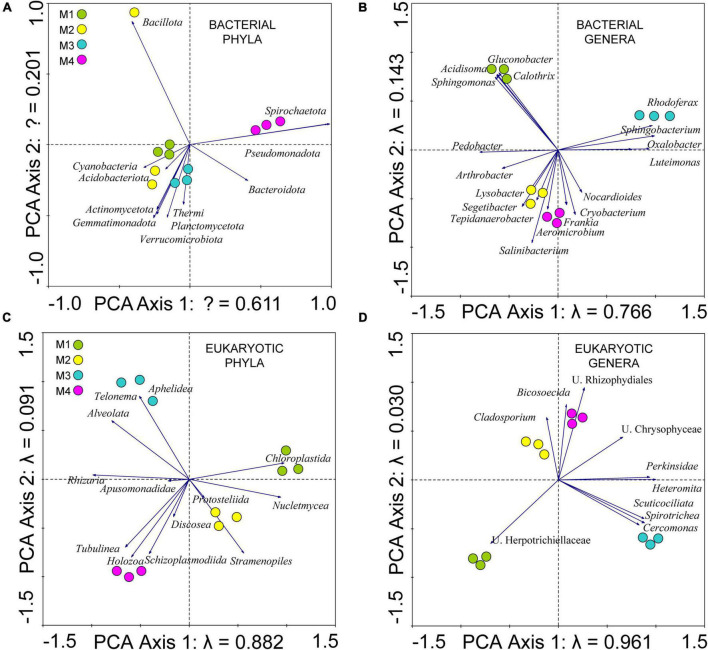
Principal component analysis (PCA). Scatterplot of **(A)** bacterial phyla, **(B)** bacterial genera, **(C)** eukaryotic phyla, and **(D)** eukaryotic genera.

To determine the distribution of microbial communities in the ice cave samples, several multivariate statistical analyses were carried out (Leps and Smilauer, 2003; Gloor et al., 2017). Given the short length of the DCA first axis gradient (not shown), the principal component analysis (PCA) was carried out (Figure 4 and Supplementary Table 4), based on the relative abundances of the microorganisms. In this analysis, the centered log-ratio transformation was used. The first axes of the PCA explained 61.1% of the total variation for bacterial phyla (Figure 4A and Supplementary Table 4 analysis no. 1), 76.6% for bacterial genera (Figure 4B and Supplementary Table 4 analysis no. 2); and 88.2 and 96.1% for microeukaryotic phyla (Figure 4C and Supplementary Table 4 analysis no. 9) and microeukaryotic genera (Figure 4D and Supplementary Table 4 analysis no. 10), respectively.

According to PCA, there was a gradient in microbial populations, which was better explained in the case of the eukaryotic community than in the case of the prokaryotes (Figures 4B, C). The first axis was mainly driven by the abundance of *Rhodoferax*, *Sphingobacterium*, *Oxalobacter*, and *Luteimonas* among bacteria, and by the genus *Heteromita* among eukaryotes. To find out which factors influence the gradient, various CCA were made using as variables both the age of the samples and the concentrations of the dissolved ions in the ice. Age explained the gradient better in the case of eukaryotes (Supplementary Figure 4 and Supplementary Table 4 analyses nos. 3, 4, 11, and 12). Ion concentrations were more closely related to the distribution of the eukaryotic community than to the prokaryotic populations (Supplementary Figures 5, 6 and Supplementary Table 4).

### Microbial activity inferred from the proteome

**FIGURE 5:**
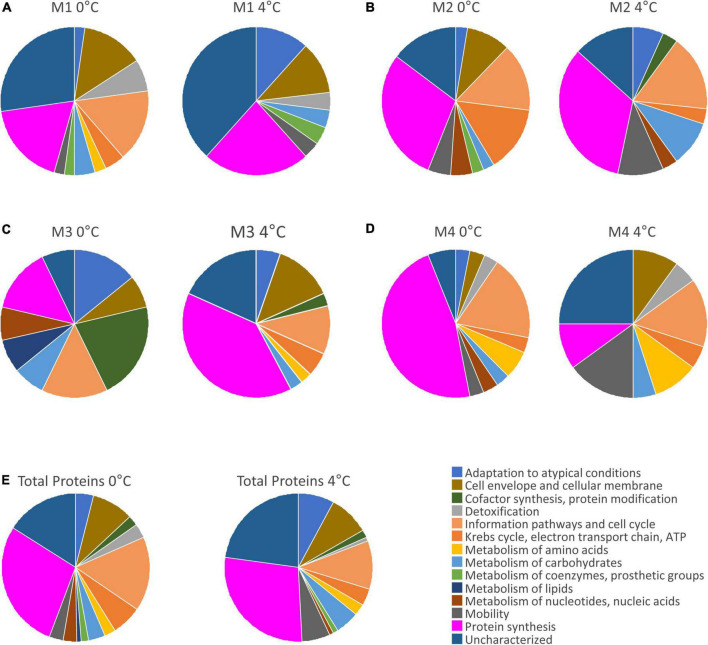
Pie charts comparing protein distribution at 0 and 4°C. **(A–D)** Pie charts corresponding to samples M1–M4, respectively. **(E)** Total protein distribution at 0 and 4°C.

The biological activity of microorganisms can be inferred from genomics, but proteomic techniques provide more reliable information because they identify the cellular machinery that is actively working, depending on environmental conditions. In this study, the metaproteomes of four samples grown at 0 and 4°C were compared to check how an increase in temperature could alter the functioning of microorganisms and to assess whether they can adapt to this state. The results of these analyses were represented in Figure 5 and Supplementary Figure 7. In general, it could be observed that all the samples (except M3) cultured at 4°C contained a lower number of total proteins (Supplementary Table 5), although the biomass had increased (Supplementary Figure 2). This may be due to a decrease in biodiversity or in protein synthesis as a result of an unfavorable situation for the microbial population.

The characteristics of each of the functional categories of proteins and examples of some of the proteins obtained in each group are extensively detailed in Supplementary discussion. The most notable differences in the number of spots between samples were observed in the “Information pathways and cell cycle” group (Supplementary Table 5). Most of them were RNA polymerases, DNA-binding response regulators and transcriptional regulators. Gene expression in bacteria begins with the promoter recognition by the DNA-dependent RNA polymerase followed by transcription initiation. The expression of many genes is subject to variation in response to environmental changes. This is achieved either through numerous mechanisms of regulation of the RNA polymerase, or by a change in the set of promoters to which the RNA polymerase can bind to Browning and Busby (2016). Although it had been described that these environmental variations could be, for example, the levels of extracellular nitrate and nitrite ions (Tyson et al., 1994), in these experiments the gene expression also varied with changes in temperature. Other identified proteins in the samples, such as relaxases, are proteins required for the horizontal transfer of genetic information contained on plasmids that occurs during bacterial conjugation (Guzmán-Herrador and Llosa, 2019).

Significant differences in the number of spots were also found in the groups “Krebs cycle, electron transport chain and ATP synthesis” and “Metabolism of nucleotides and nucleic acids” (Supplementary Table 5). The number of proteins was higher in the samples grown at 0°C. These proteins were mainly kinases, ligases, syntases, and oxidoreductases. A predominance of these types of enzymes had also been reported in the cold adaptation of the psychrophilic bacterium *Pseudoalteromonas haloplanktis* (Margesin et al., 2003; Wilmes et al., 2011).

The samples incubated at 4°C presented a greater number of spots related to motility such as flagellin, tail protein, chemotaxis protein, and motor switch protein. When temperature rises, the ice melts and the liquid medium in veins between ice channels facilitates the movement of the cells (García-Descalzo et al., 2022).

Once the functions of the proteins were identified, the results obtained in each sample at 0 and 4°C could be compared. These differences could be observed in the sector diagrams (Figures 5A, D). From these data, it could be deduced that the microorganisms present in the M1 and M2 samples did not withstand the increase in temperature. Also, the microorganisms in the M3 sample were altered by the increase in temperature. For M4, it is remarkable that the two diagrams were very similar (Figure 5D), especially regarding the relevant categories to assess their adaptation. In this case, the adaptation to the increase in temperature was much better than in the other samples.

### Biogeochemical cycles inferred from taxonomy and proteomics

**FIGURE 6:**
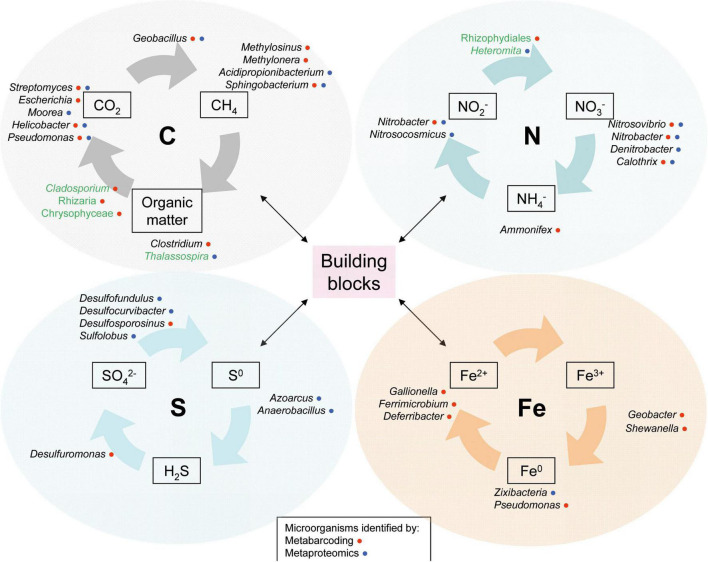
Model of the metabolic potentials between dominant microorganisms in the ice cave. Some key members were represented at genus level (Prokaryotes in black, Eukaryotes in green).

The so-called biogenic elements, together with other minor elements, are combined in living organisms to form the primary monomers or “building blocks” of life: sugars, amino acids, nucleotides, and lipids. The actions of the microorganisms that allow the recycling of these main elements are fundamental for the correct functioning of the ecosystem to which they belong. In this study, microorganisms identified in the taxonomic study and by proteomics participate in the biogeochemical cycles of carbon, nitrogen, sulfur, and iron. This information was summarized in Figure 6.

The identified microorganisms that participate in the carbon cycle obtain this element from either atmospheric CO_2_ from the carbon-rich karstic rocks that form the cave; and by decomposing organic remains, mainly leaves or branches, trapped in the ice. The processes involved in this cycle are widely known, but photosynthesis, capable of transforming CO_2_ into organic matter (for example, algae belonging to the *Chrysophyceae* class), along with respiration or fermentation, which oxidize organic matter to CO_2_, stand out. Also relevant in this system is the action of chemolithotrophic organisms, responsible for assimilating the carbon available in the rocks (*Streptomyces*), is relevant. Additionally, the presence of methylotrophs was detected, which under aerobic conditions catabolize C1 compounds such as CH_4_ (*Methylosinus* or *Methylotenera*). Several proteins that take part in the carbon metabolism, such as the alpha-L-fucosidase (*Sphingobacterium*), alpha-amylase (*Acidipropionibacterium*), pyruvate dikinase (*Helicobacter*), fructose-bisphosphatase (*Pseudomonas*), acetaldehyde dehydrogenase (*Geobacillus*), and alcohol dehydrogenase (*Moorea*) were too identified in the system. Among microeukaryotes, the enzyme orotidine-5′-phosphate decarboxylase from the diatom *Thalassospira* was found (Figure 6).

Several processes are carried out within the nitrogen cycle, the most important being nitrification (oxidation of NH_4_^+^ or NO_2_^–^ to NO_3_^–^), denitrification (reduction of NO_3_^–^ to N_2_ or N_*x*_O, which reduces the amount of fixed nitrogen), nitrogen fixation (assimilation of atmospheric N_2_ so that it can be used by organisms), ammonification (transformation of NH_2_ from protein groups to NH_4_^+^), and anammox (NO_2_^–^ and NH_4_^+^ are converted into gaseous N_2_). Although most of the nitrifying bacteria are strict aerobes, NH_3_ can also be oxidized under anoxic conditions in a process known as anammox. In the anammox reaction, ammonia is oxidized with NO^2–^ as the electron acceptor to yield N_2_. This process is carried out by some members of the *Planctomycetota* phylum which constitute an unusual group of obligately anaerobic Bacteria. In our study some of these bacteria were identified such as *Scalindua brodae* (Schmid et al., 2003).

Some examples of microorganisms that take part in the nitrogen cycle are: *Nitrobacter* for nitrification, *Pseudomonas* or *Bacillus* for denitrification, and *Cyanobacteria* or *Rhizobiales* for nitrogen fixation. In proteomics experiments, some proteins of participants in the nitrogen cycle such as *Nitrosocosmicus* and *Nitrobacter* were found. As the ice melts and turns into water, more carbon could be fixed and the carbon cycle would increase. In areas with less oxygen, the processes of the nitrogen cycle would be favored, promoting denitrification and the anammox process, facilitating the synthesis of NO_2_, which is a greenhouse gas (Madigan et al., 2015).

Sulfur is an element with many oxidation states, although only three of them have biological importance (S^0^, S^2–^, and S^6+^). This data and the fact that some parts of the cycle are carried out in abiotic situations, make this cycle more complex. Sulfur can be found naturally in minerals and as gaseous SO_2_ from volcanic eruptions. Some examples of microorganisms that take part in this cycle could be *Thiobacillus*, a sulfur chemolithotroph capable of oxidizing H_2_S or S^0^ compounds to SO_4_^2–^; *Desulfovibrio* or *Desulfobacter*, which anaerobically reduce SO_4_^2–^ to H_2_S; and *Desulfuromonas*, responsible for reducing S^0^ to H_2_S. Sulfur assimilation is essential for the synthesis of amino acids with -S- (methionine) and -SH (cysteine) groups.

Iron is one of the most abundant elements in the earth’s crust and is an element present in living beings, although in less quantity than in the previous cases. Some representatives of the sulfur cycle were identified through their proteins, for example *Azoarcus*, *Desulfofundulus*, *Sulfolobus*, *Desulfocurvibacter*, and *Anaerobacillus alkalidiazotrophicus*.

In the biogeochemical cycle of iron, this element only exists in 3 oxidation states. In this study, some microorganisms of the genera *Gallionella* and *Ferrimicrobium*, which are involved in oxidation processes of Fe^2+^ to Fe^3+^, and other genera such as *Ferrireducens*, that reduces Fe^3+^ to Fe^2+^, were identified.

In addition to these biogeochemical cycles, microorganisms that participate in other important metabolic processes were isolated, such as the microeukaryotes *Cladosporium*, which oxidize manganese and absorb cadmium; or bacteria such as *Pseudomonas*, *Burkholderia*, and *Enterobacter*, that are involved in the phosphorus cycle.

## Conclusion

This study provides information on the hidden microbial ecosystem in the perennial ice from a Pyrenean ice cave. The results (i) gave important information about a hidden ecosystem that is under imminent risk of disappearing; (ii) simulated the *in vivo* effect of climate change in a frozen cave, and how a 4°C rise in temperature would affect its microbial populations; (iii) identified the prokaryotic and eukaryotic microbial communities that inhabit a frozen cave in the Pyrenees; (iv) studied the molecular modifications that affect the cellular machinery of these microorganisms; (v) found several proteins and enzymes that show adaptation of cells to a higher temperature; (vi) documented the influence of climate change on biogeochemical cycles; (vii) identified several proteins that are expressed at higher temperatures and could be considered sensors of climate change.

The microorganisms that inhabit remote ecosystems are poorly understood. Further research is needed on the identification of genetic sequences and the unknown and hypothetical proteins they encode. These proteins and enzymes have applications in various industries and are also indicators of the climatic changes to which ecosystems are undergoing.

## Data availability statement

Sequences obtained by 16S rRNA and 18S rRNA sequencing have been deposited in NCBI Short Read Archive (SRA) (accession number PRJNA663780). The sequences obtained by 18S rRNA sequencing were also deposited in the European Nucleotide Archive (ENA) (https://www.ebi.ac.uk/ena/submit/sra/). The assigned ENA accession number was PRJEB32471. The mass spectrometry proteomics data are deposited to the ProteomeXchange Consortium via the PRIDE repository with the dataset identifiers PXD029615, PXD033406, and PXD033535.

## Author contributions

CC and AM designed the study and wrote the manuscript. AM led the scientific project. EG-L, CC, ML, VM-H, and FR-B generated the data and conducted the analysis. CC, MB, and ML participated in the field campaigns. All authors participated in the interpretation of the results and reviewed, commented, and edited the manuscript.

## Footnotes

1
http://proteomecentral.proteomexchange.org

2
www.graphpad.com
