# Supplementary material for: The hidden microbial ecosystem in the perennial ice from a Pyrenean ice cave
Source: Front Microbiol. 2023 Jan 26;14:1110091. doi: 10.3389/fmicb.2023.1110091 (PMC9909108; doi:10.3389/fmicb.2023.1110091)
Supplement: Supplementary file 1 [file Data_Sheet_1.pdf]

SUPPLEMENTARY INFORMATION FOR:

The hidden microbial ecosystem in the perennial ice from a Pyrenean ice-cave

Fátima Ruiz-Blas[1,2], Víctor Muñoz-Hisado[1], Eva Garcia-Lopez[1], Ana Moreno[3], Miguel Bartolomé[3,4], Maria Leunda[5,6,7], Emma Martinez-Alonso[8], Alberto Alcázar[8], and Cristina Cid[1]*

1 Centro de Astrobiologia (CAB), CSIC-INTA, Carretera de Ajalvir km4, 28850 Torrejón de Ardoz, Madrid, Spain
2 GFZ German Research Centre for Geosciences, Section Geomicrobiology, Telegrafenberg, 14473 Potsdam, Germany
3 Departamento de Procesos Geoambientales y Cambio Global, Instituto Pirenaico de Ecología - CSIC, 50059, Zaragoza, Spain
4 Institut für Geologie und Mineralogie, Universität zu Köln, Zülpicher Strasse 49b, 50674, Köln, Germany
5 Institute of Plant Sciences & Oeschger Centre for Climate Change Research. Altenbergrain 21, 3013 University of Bern, Bern, Switzerland
6 Swiss Federal Research Institute for Forest, Snow and Landscape Research WSL
Zürcherstrasse 111 8903 Birmensdorf, Zürich, Switzerland
7 Department of Plant Biology and Ecology, University of the Basque Country, Leioa, Spain
8 Department of Investigation, Hospital Ramón y Cajal, Instituto Ramón y Cajal de Investigación Sanitaria, 28034 Madrid, Spain

* Corresponding Author: cidsc@inta.es

This file includes:

I. Supplementary Tables S1-S5

II. Supplementary Figures. Figures S1-S7

III. Supplementary Discussion

IV. Supplementary References

I. Supplementary Tables S1-S5

Table S1. Culture media.

	**Culture media (g/L)**
**T2** (20x)	5 g peptone, 0.15 g ferric ammonium citrate, 0.2 g $MgSO_4 \cdot 7H_2O$, 0.05 g $CaCl_2$, 0.05 g $MnSO_4 \cdot H_2O$, 0.01 g $FeCl_3 \cdot 6H_2O$
**T3** (20x)	1 g glucose, 1 g peptone, 0.5 g yeast extract, 0.2 g $MgSO_4 \cdot$7H2O, 0.05 g $MnSO_4 \cdot 4H_2O$
**T4** (20x)	1 g glucose, 0.5 g casamino acids, 0.5 g yeast extract, 1 g KH_2PO_4, 0.5 g $CaCl_2 \cdot 2H_2O$, 0.5 g $MnCl_2 \cdot 4H_2O$
**T5** (20x)	R2A 20x
**T6** (1x)	R2A
**T7** (20x)	Marine broth 2216
**T8** (20x)	Trypticase soy broth
**T9**	Ice meltwater

Table S2. Analysis of bacterial 16S rRNA
File: Table S2.xlsx.

Table S3. Analysis of eukaryotic 18S rRNA
File: Table S3.xlsx.

Table S4. Correspondence analyses and species-environment correlations (λ).

No. of analysis	Type of microorganism	Level	Type of analysis	Environmental variables	λ_1	λ_2	λ_3	λ_4	Figure
1	Bacteria	Phylum	PCA	-	0.611	0.201	0.165	0.023	4A
2		Genus	PCA	-	0.766	0.143	0.089	0.001	4B
3		Phylum	CCA	Age	0.034	0.070	0.028	0.018	S2A
4		Genus	CCA	Age	0.261	0.216	0.163	0.002	S2B
5		Phylum	CCA	NH_4^+, NO_2^-, NO_3^-, SO_4^{2-}, SRP, DOC	0.061	0.047	0.028	0.003	S3A
6		Genus	CCA	NH_4^+, NO_2^-, NO_3^-, SO_4^{2-}, SRP, DOC	0.389	0.167	0.086	0.000	S3B
7		Phylum	CCA	C, Na, Mg, Al, Si, P, S, K, Ca, Mn, Fe, Cu, Zn, As, Br, Sr, Sn, I	0.072	0.048	0.028	0.003	S4A
8		Genus	CCA	C, Na, Mg, Al, Si, P, S, K, Ca, Mn, Fe, Cu, Zn, As, Br, Sr, Sn, I	0.390	0.167	0.086	0.000	S4B
9	Eukarya	Phylum	PCA	-	0.882	0.091	0.027	0.000	4C
10		Genus	PCA	-	0.961	0.030	0.010	0.000	4D
11		Phylum	CCA	Age	0.197	0.064	0.042	0.001	S2C
12		Genus	CCA	Age	0.218	0.236	0.098	0.001	S2D
13		Phylum	CCA	NH_4^+, NO_2^-, NO_3^-, SO_4^{2-}, SRP, DOC	0.255	0.045	0.003	0.000	S3C
14		Genus	CCA	NH_4^+, NO_2^-, NO_3^-, SO_4^{2-}, SRP, DOC	0.412	0.099	0.041	0.000	S3D
15		Phylum	CCA	C, Na, Mg, Al, Si, P, S, K, Ca, Mn, Fe, Cu, Zn, As, Br, Sr, Sn, I	0.255	0.046	0.003	0.000	S4C
16		Genus	CCA	C, Na, Mg, Al, Si, P, S, K, Ca, Mn, Fe, Cu, Zn, As, Br, Sr, Sn, I	0.413	0.099	0.041	0.000	S4D

Table S5. Identification of proteins isolated from the Pyrenean ice cave A294
File: Table S5.xlsx. (Proteomic dataset file)

Abbreviations used: Theoretical molecular weight (ThMw), theoretical isoelectric point (Th pI), number of mass values searched/number of mass values matched (N), % sequence coverage (C). Protein scores higher than 66 were significant ($p<0.05$) in the Mascot database search algorithm (Matrix science, UK).

II. Supplementary Figures. Figures S1-S7

Figure S1. Summary of the overall experimental strategy.

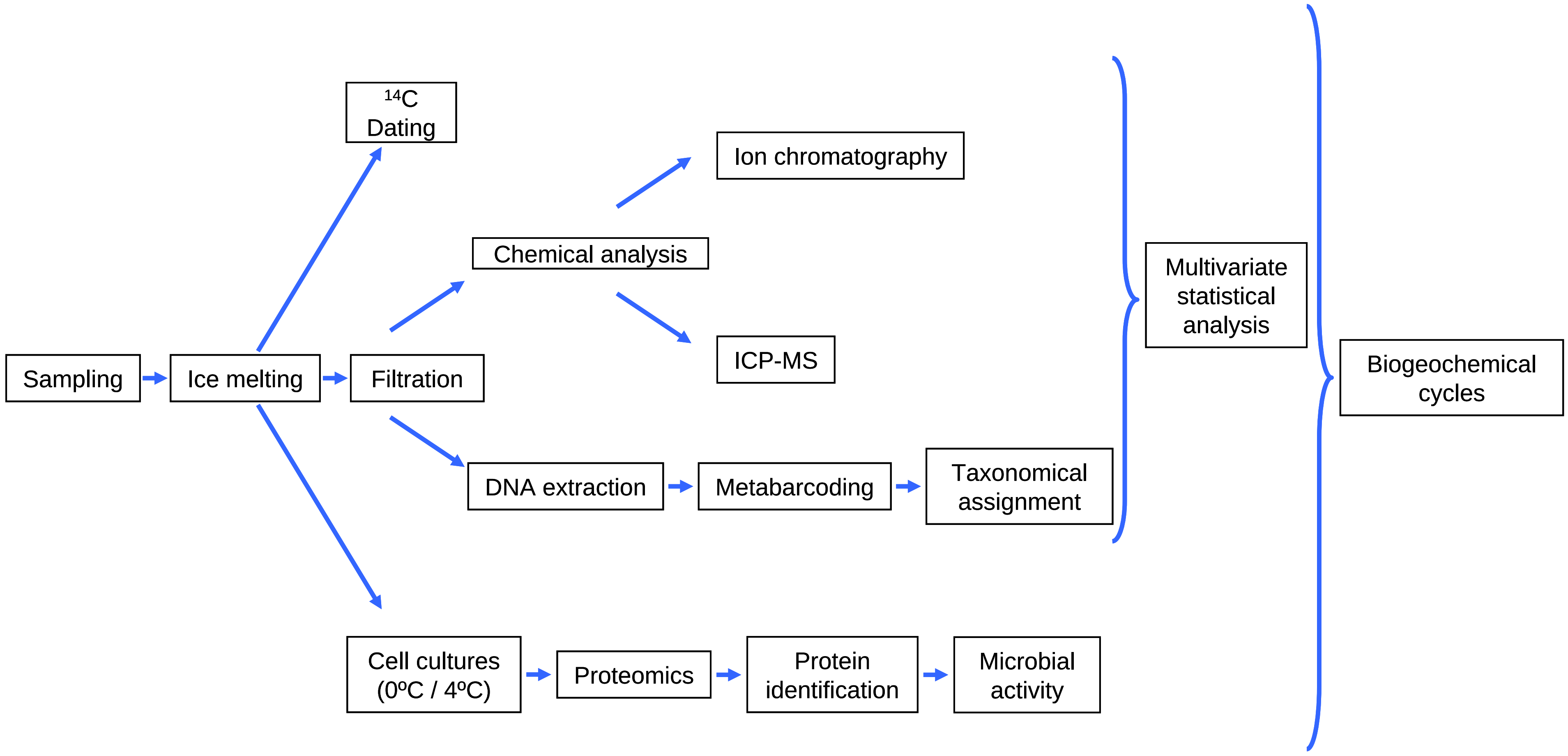

Figure S2. Growth rates monitored by optical density at 600 nm. The data shown are the mean ±SD of 3 cultures for each culture condition.

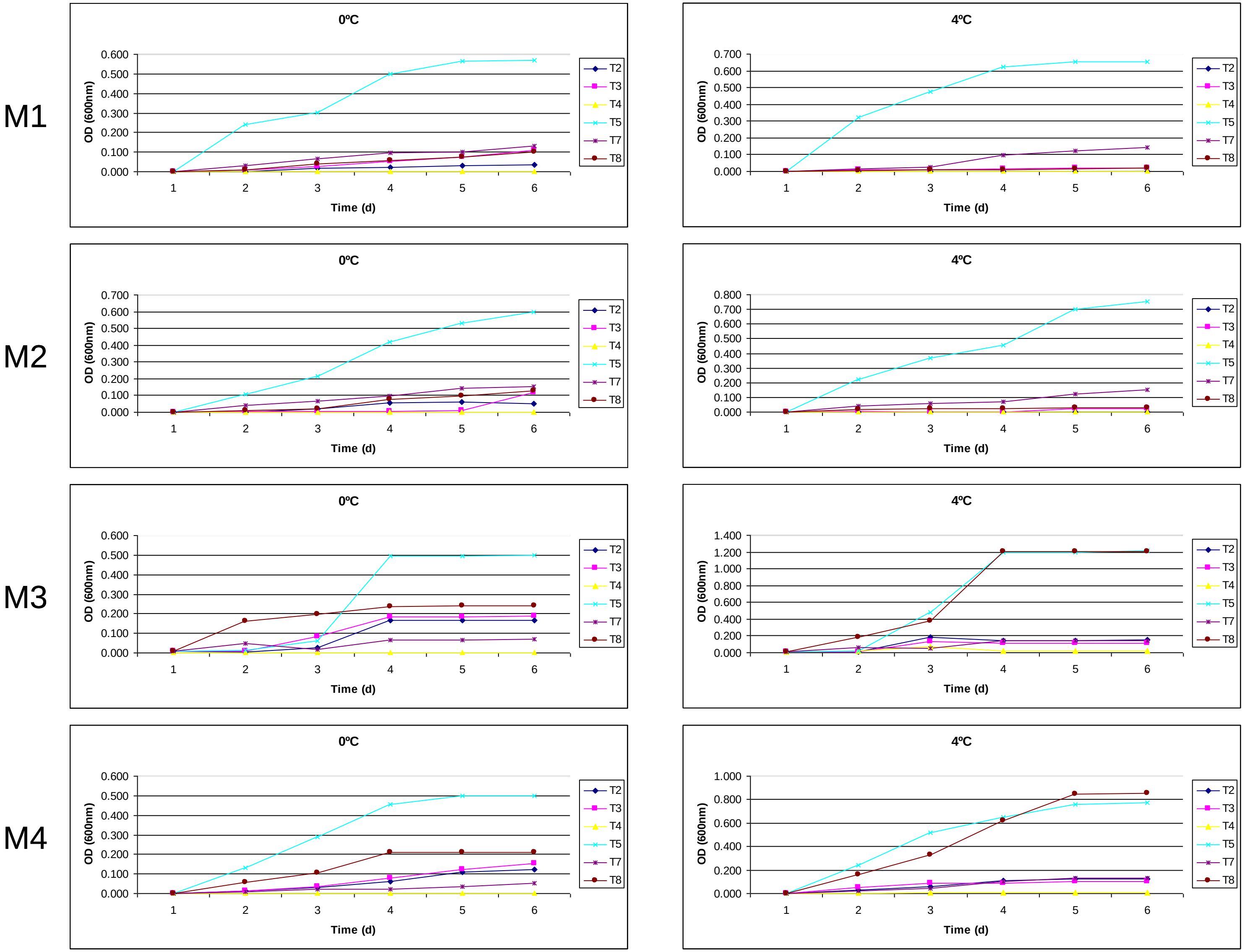

Figure S3. Relative abundance (No. reads) of to the most abundant genus in each sample.

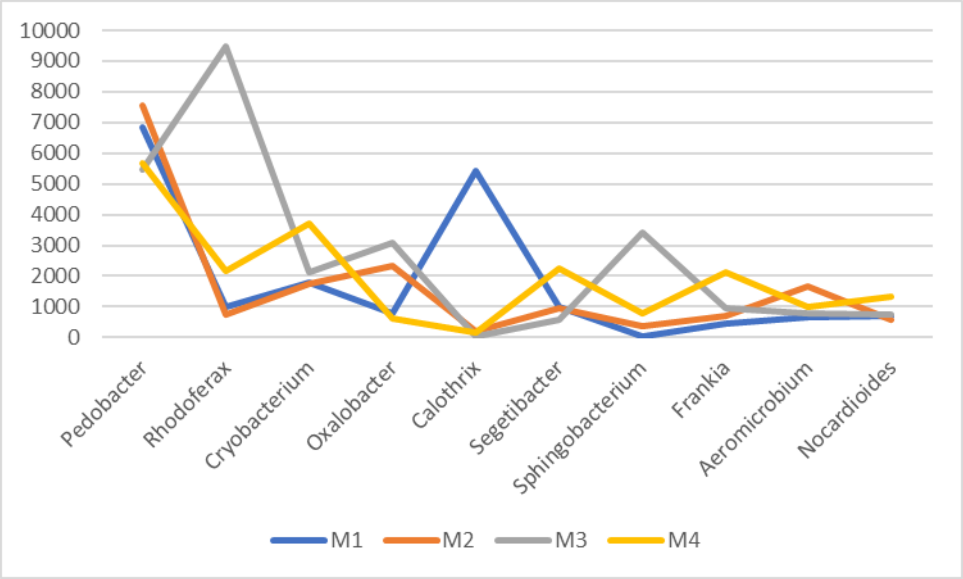

Figure S4. CCA of the microbial groups with regard to the ice age. Analysis of (A) bacterial phyla, (B) bacterial genera, (C) eukaryotic phyla, and (D) eukaryotic genera.

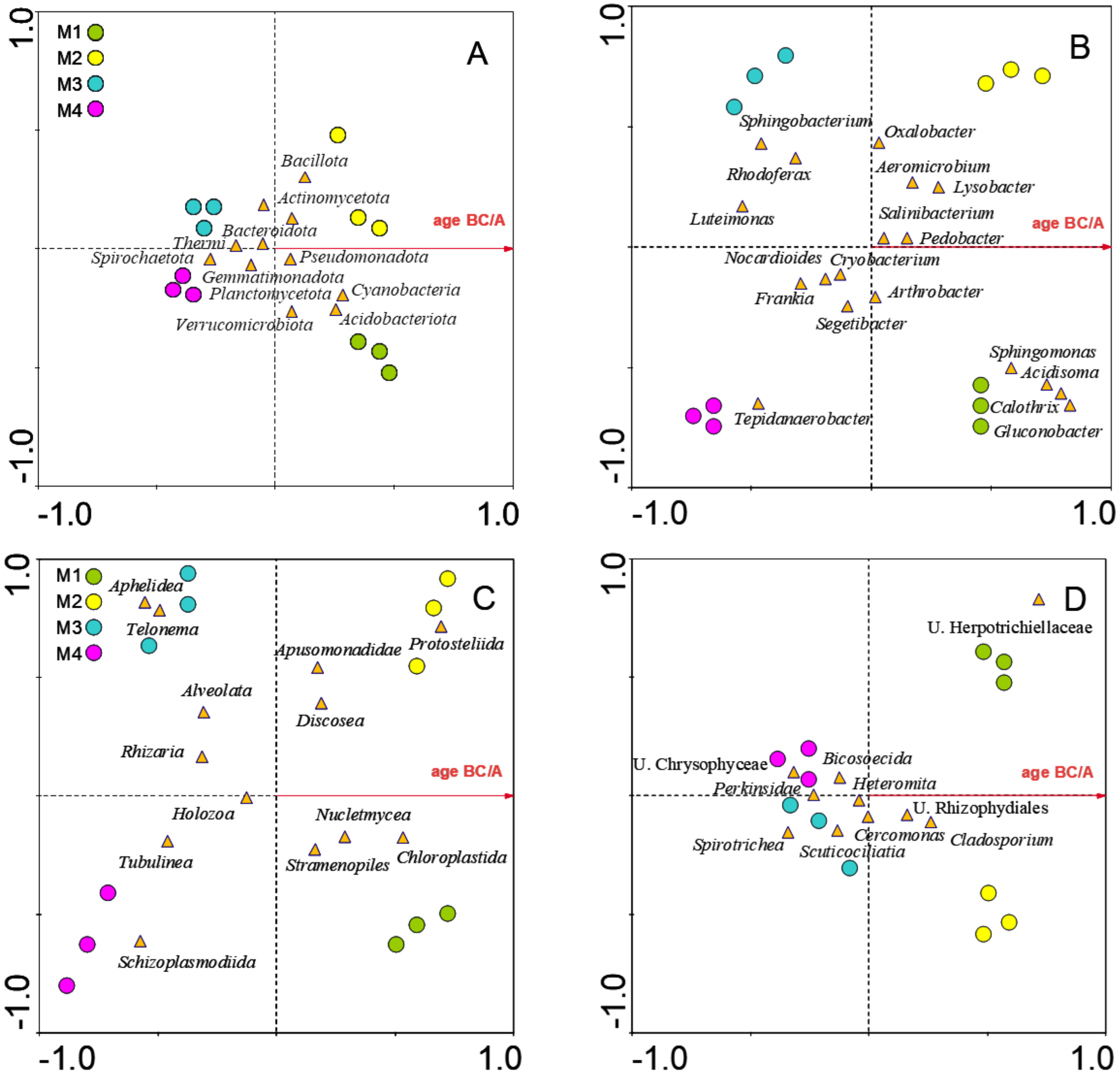

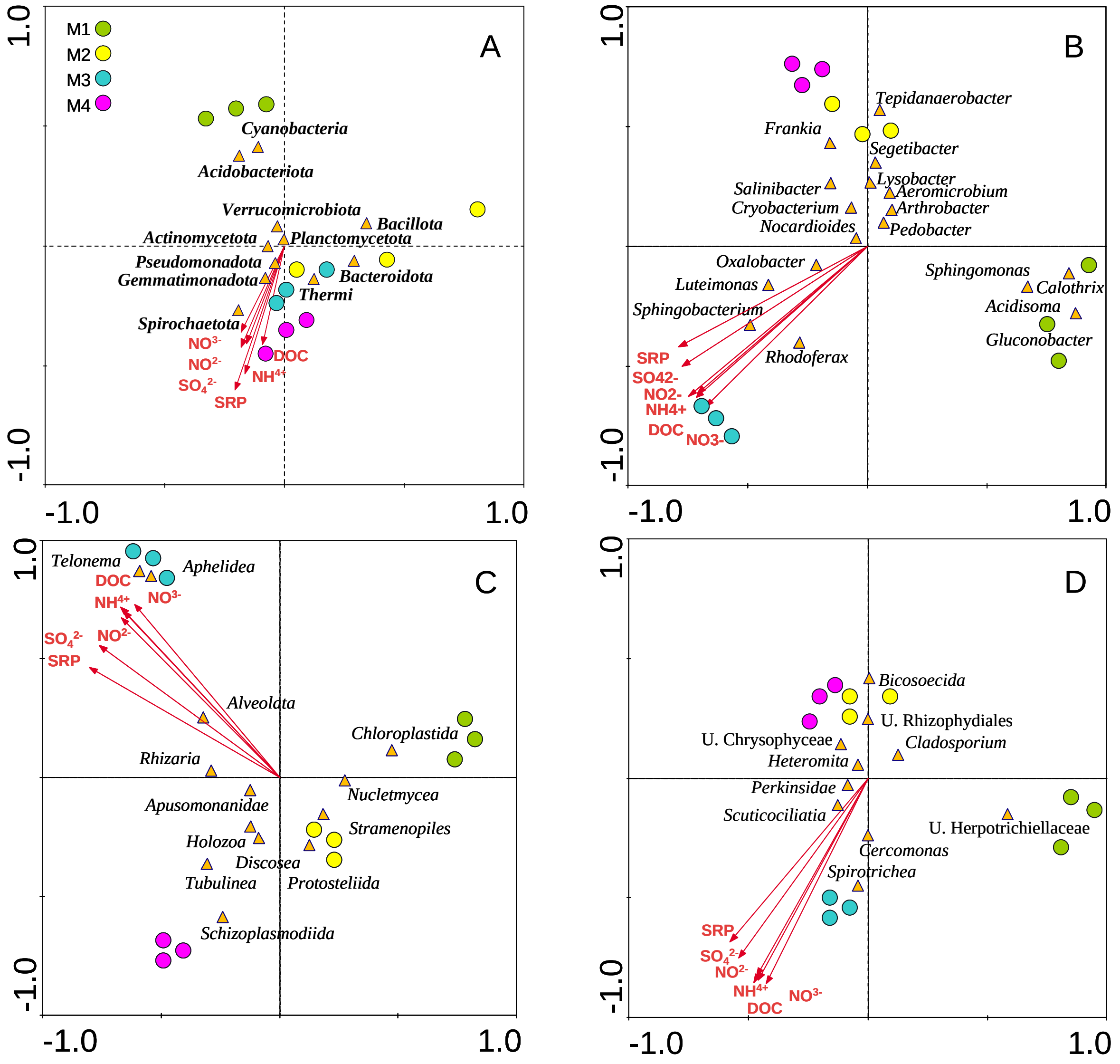

Figure S5. CCA of the microbial groups with regard to the nutrient concentrations. Analysis of (A) bacterial phyla, (B) bacterial genera, (C) eukaryotic phyla, and (D) eukaryotic genera.

Figure S6. CCA of the microbial groups with regard to the concentrations of the dissolved ions in the ice. Analysis of (A) bacterial phyla, (B) bacterial genera, (C) eukaryotic phyla, and (D) eukaryotic genera.

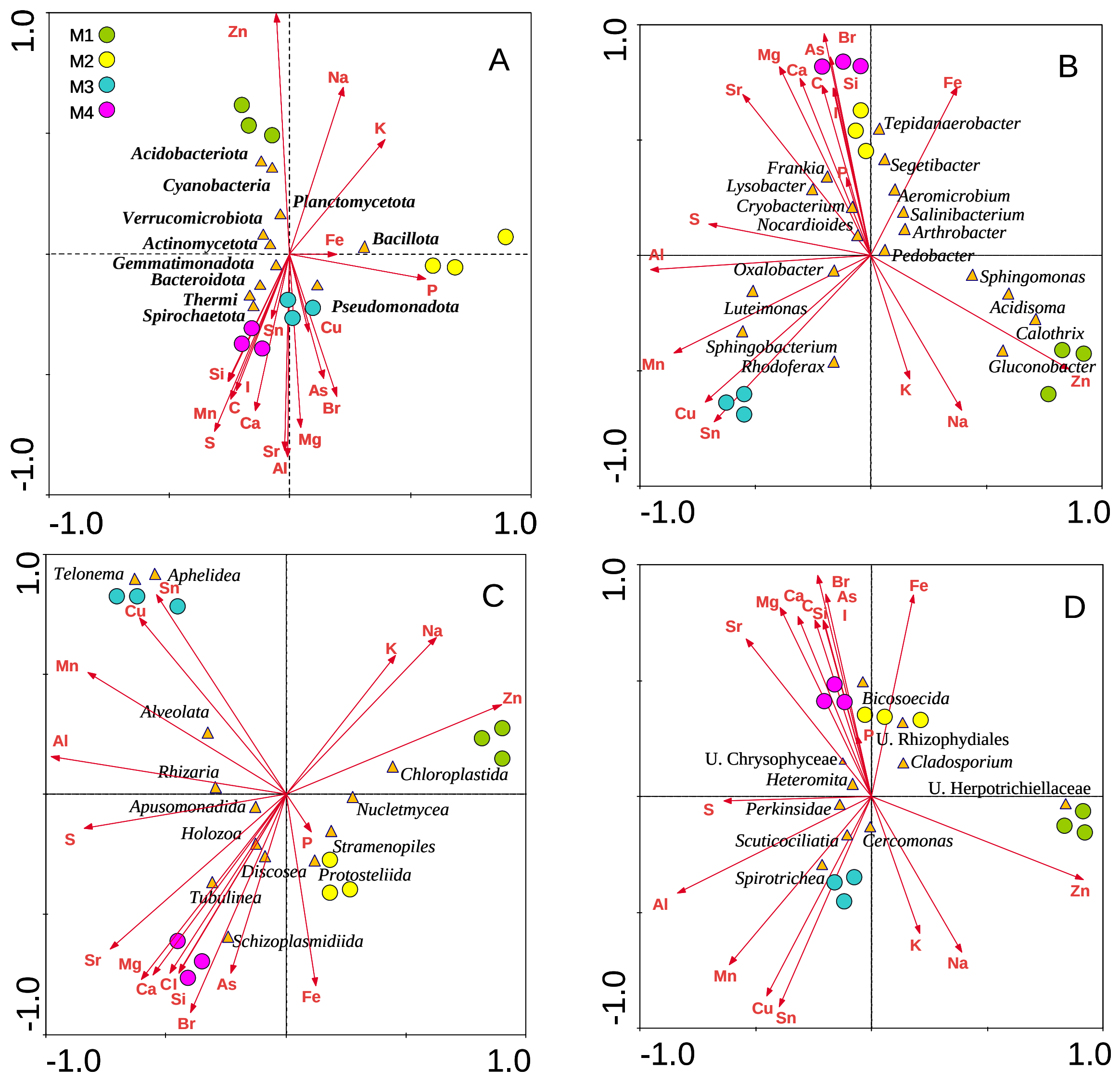

Figure S7. Soluble proteins from glacier samples and from cultures resolved by 2-DE. Numbered spots marked with circles corresponded to proteins identified by MALDI-TOF and described in Table S4. The figure is representative of four 2-DE experiments.

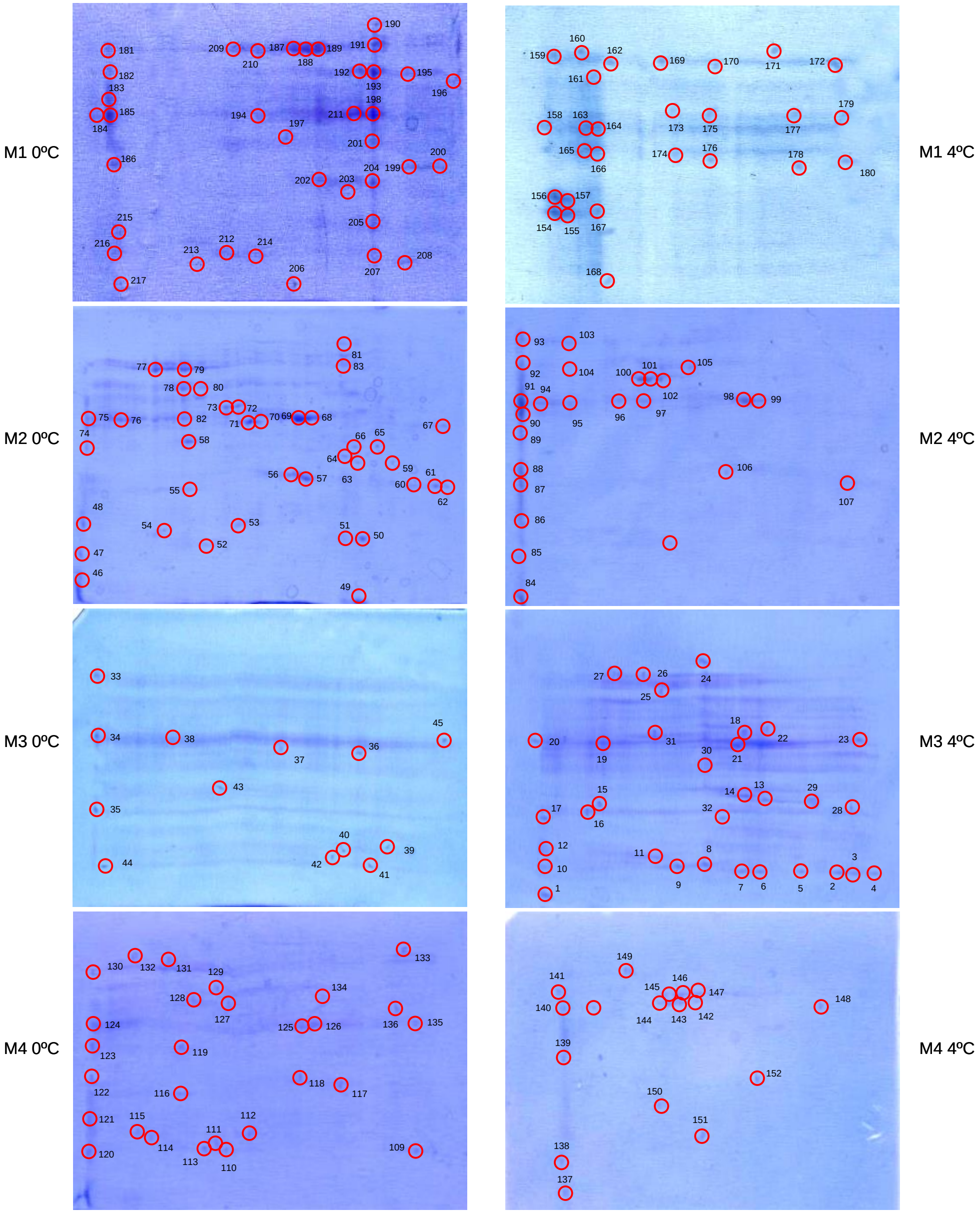

III. Supplementary Discussion

The characteristics of each of the functional categories are detailed below, and examples of some of the proteins obtained in each group are given.

Adaptation to atypical conditions

The proteins in this category have functions related to the adaptation of microorganisms to stress conditions that can induce the death of cells. Proteins involved in the sporulation process are also included in this group since spore formation can be a stress response.

This kind of proteins appeared in both proteomes, although they were a majority in the cultures carried out at 4ºC. An example could be the ATP-binding subunit of the ATP-dependent endopeptidase Clp that appeared in the proteome obtained from the M3 sample when cultured at 4ºC. Its function is to use the energy from hydrolyzing ATP to degrade misfolded proteins, acting in a similar way to chymotrypsin (Zellmeier et al., 2006).

The AAA+ family of ATPases also appeared on several occasions. They have various functions, including the regulation of the cell cycle, the participation in proteolysis or protein breakdown, and act also as chaperones. Within this family, some proteins take part, together with the DnaK, in the response to hyperosmosis and heat shock. Among them, the heat shock proteins, or HSPs proteins prevent the aggregation of proteins denatured by stress (Mondini et al., 2022). In our samples, this category is more abundant in samples at 4ºC than in samples at 0ºC. This fact indicates that the microorganisms are living in a stressful condition at 4ºC.

Another chaperone, the GroEL, was identified from *Pseudomonas* and *Massilia*, this protein contributes to the correct folding of proteins and does also protect the cell from harmful situations (García-Descalzo et al., 2014).

Krebs Cycle, Electron Transport Chain and ATP Synthesis

The proteins involved in the Krebs cycle metabolize carbohydrates, lipids and proteins that cells use to obtain energy in the form of GTP under aerobic conditions, while those involved in the electron transport chain create an electrochemical gradient that is used to synthesize ATP that will later be used in cellular processes as a source of energy.

The presence of proteins in this category, together with other types of enzymes that degrade carbohydrates, lipids and proteins to molecules that serve as substrates for enzymes involved in the Krebs cycle, implies that the cells are obtaining energy to carry out the processes that allow them to fulfill vital functions, such as reproduction, or relationship with their environment. Therefore, the fact that this group of proteins is more numerous at 0ºC implies that under these conditions the microorganisms can live more comfortably. At 4ºC energy is also produced using these proteins, but to a lesser extent.

As an example, the enzyme succinate-CoA ligase (both alpha and beta subunits) which takes part in the Krebs cycle, has been identified in sample M2. This enzyme participates in the only step of the tricarboxylic acid cycle in which phosphorylation occurs at the substrate level, where succinate-CoA becomes succinate, and at the same time ADP is phosphorylated, becoming ATP (Joyce et al., 1999).

Regarding the enzymes involved in the electron transport chain and the synthesis of ATP, proteins such as ATP synthases or oxidoreductases repeatedly appeared in gels. In bacteria, this process is carried out through an electrochemical gradient generated on both sides of the membranes, as it occurs in eukaryotes. The main difference is that bacteria are able to adapt more easily to extreme situations since they contain a larger number of electron donors and acceptors.

Detoxification

This category includes proteins that degrade certain molecules that can be harmful to the cell. They are responsible for removing heavy metals, although they mainly act as antioxidants. Examples include the thiol peroxidase enzyme, isolated in the proteome of M1 at 4ºC; or the enzyme peroxiredoxin isolated in the proteome of M4 at 0ºC. This enzyme, is oxidized by hydrogen peroxide, reducing it and preventing it from generating oxidative stress in the cell, thus becoming an antioxidant enzyme. It is also related to the activation of certain genes (Jeong et al., 2000).

Metabolism of amino acids and related molecules

The proteins classified in this category have functions related to the degradation of proteins and amino acids, used to later synthesize new proteins. Amino acids are broken down because they are not needed or because the cell needs energy and cannot get it from the main pathways of carbohydrate metabolism. In this category, the same number of total proteins was obtained in the proteome at 0ºC and 4ºC (Table S5). It can then be confirmed that when the temperature increases, bacteria continue to carry out these processes in a similar way as they do under standard conditions since they are common to both proteomes.

An example would be the protein Saccharopine dehydrogenase, obtained in the M4 proteome at 0ºC, which catalyzes the NAD+-dependent reaction in which L-Saccharopine is hydrolyzed to L-lysine (an amino acid necessary for protein synthesis) and 2-oxoglutarate (an intermediate of the Krebs cycle) (de Mello Serrano et al., 2012).

Metabolism of carbohydrates and related molecules

The proteins included in this group have functions related to the degradation of carbohydrates to obtain energy in the form of ATP that cells can use to carry out various processes necessary to survive. Therefore, the presence of these proteins indicates that the cell is functioning optimally since it is producing energy to develop its basic functions.

This is not a numerous group of proteins, but was present in both the proteomes obtained at 0ºC and 4ºC. This fact could indicate that the bacteria that are synthesizing these proteins are adapting correctly to the increase in temperature.

An example is the fructose bisphosphatase class 1, also known as fructose-1,6-bisphosphatase, which appeared repeatedly in gels from cultures at both 0ºC and 4ºC, and catalyzes the hydrolysis of fructose-1,6-bisphosphate to fructose-6-phosphate during the gluconeogenesis. (Hines et al., 2007).

Metabolism of coenzymes and prosthetic groups

The proteins contained in this category are related to the metabolism and synthesis of coenzymes and prosthetic groups. It is not very numerous in either of the two conditions, but its presence is necessary for the correct functioning of the cell.

One of the proteins that appeared in this group was the enzyme exopolyphosphatase from the chemolithoautotrophic bacteria *Bosea lupini*. This enzyme is capable of degrading inorganic polyphosphates because it hydrolyzes the terminal phosphate groups of a polyphosphate chain, thus leaving free phosphate anions that the microorganism can use (Beassoni et al., 2015).

Metabolism of nucleotides and Nucleic Acids

The proteins included in this category participate in processes related to the synthesis and degradation of nucleotides and nucleic acids. Although it is not a very large group, a higher number of proteins from this class were isolated from the proteomes of bacteria grown at 0ºC than from the proteomes of bacteria grown at 4ºC. As mentioned above, the bacteria must synthesize proteins that help to obtain ATP, since this ATP is then used as a source of energy to carry out all cellular processes.
The enzyme carbamate kinase, identified in the M2 proteome at 0ºC, catalyzes the transfer of a phosphate group from an ATP to hydrogen carbonate to obtain ADP and carbamoyl phosphate. It is part of the carbamoyl phosphate degradation pathway, in which this compound is degraded to give CO_2 and NH_3 (Ramón-Maiques et al., 2010).

Metabolism of Lipids
The proteins included in this category have functions related to the degradation and synthesis of lipids. Although this group is not numerous and was only isolated in one of the proteomes obtained at 0ºC, this does not mean that it was not present in the rest of the conditions, since they are necessary, and complement some of those obtained in other more abundant categories. Probably they were not obtained in the rest of the proteomes because the level of expression was lower.
The only identified enzyme belonging to this category, the enoyl-CoA hydratase from *Bradyrhizobium*, was isolated in the M3 proteome at 0ºC. This enzyme transforms a double bond into a single bond with a hydroxyl radical by adding a water molecule. This process is part of the beta-oxidation of fatty acids, which is the process by which fatty acids are transformed into acetyl-CoA to enter the Krebs cycle and thus obtain energy (Fukui, et al., 1998).

Mobility
Some of the isolated species can form a flagellum that helps them move through the ice channels. These proteins were more abundant in the case of the proteomes obtained at 4ºC, which could be because at this temperature the ice is partially melted and waterways are formed in which the microorganisms can move.
The most abundant protein among those belonging to this category is flagellin identified in *Pseudomonas*, a protein capable of polymerizing to form filaments or a bacterial flagellum (Titz et al., 2006).

Cell wall and membrane processes
The proteins from this category have functions related to the formation of the bacterial peptidoglycan wall that protects the bacteria and other cellular processes associated with the construction of the plasma membrane.
It was one of the most abundant categories in both conditions since it encompasses proteins that are involved in the transport of substances through the membrane, sensors of some stimulus, and cell mechanisms to defend themselves against bacteriophages or proteins that are involved in the synthesis of peptidoglycans.
An example is D-alanine-D-alanine ligase, which bonds two molecules of alanine thanks to the hydrolysis of ATP. This reaction is basic in the biosynthesis of peptidoglycans, which are then used to form the cell wall (al-Bar et al., 1992).
Other proteins that also belong to this category are the ABC transporters, which can transport substances through the membrane using the energy they obtain from the hydrolysis of ATP.

Synthesis of cofactors and protein modifiers
The proteins in this group develop functions related to the synthesis of cofactors or protein post-translational modifiers. This category is closely related to the *Protein synthesis* group, although this was less abundant since only some proteins need cofactors or modifiers.
An example is the SAM protein from the methanotroph *Roseiarcus fermentans* isolated in the proteome of M3 at 0ºC. This superfamily takes its name from S-adenosyl-L-methionine (SAM). A type of a protein belonging to this family is Ubiquinone/menaquinone C-methyltransferase, which participates in the biosynthesis of these cofactors.

Protein synthesis
This was the largest category in both cases, although it was slightly superior in proteomes obtained from cultures grown at 0ºC. Protein synthesis only occurs in living cells, and their presence indicates that these microorganisms are capable of living inside the frozen cave. The proteins synthesized by cells are used for the development of their vital functions and also to adapt to stress situations.
For instance, the elongation factors G, Ts and Tu, were isolated in almost all of the proteomes at 0ºC and 4ºC. These proteins aim to lengthen the amino acid chain during protein translation (Doherty et al., 2006).

Information pathways and cell cycle
The presence of proteins belonging to this group indicates both the existence of cell division, as DNA replication is taking place and the expression of the bacterial genes through the transcription of DNA to RNA, that leads to protein synthesis.
This category was one of the most numerous in both conditions, but was significantly more abundant (close to double) in the proteomes obtained from cultures grown at 0ºC (Table S5). These proteins play a key role in the development of the cell and the growth of the colony. The reason for this large difference could be that bacteria grown at 4ºC are devoting more means to adapting to unfavorable conditions than to reproduction, and the growth of bacteria would also be reduced.
In addition, as the number of proteins dedicated to protein synthesis is similar in both conditions, the difference in this category could be due to the difference in the processes of reproduction and the cell cycle, which occurred to a greater extent in cultures at 0ºC.
An example of isolated proteins involved in the cell cycle would be cell division protein ZapA, which was obtained in the M4 proteome at 4°C from *Paenibacillus*. This protein activates cell division by inhibiting the GTPase activity of FtsZ, allowing the recruitment of the protofilaments that form the Z ring (Johnson et al., 2004).
Several proteins were also observed in almost all of the proteomes at 0ºC and 4ºC that were involved in the transmission of genetic information, such as DNA polymerases or RNA polymerases, which participate in the processes of replication and transcription.

Uncharacterized
This category includes undetermined proteins, hypothetical proteins, and DUF (domain of unknown function) proteins. Although their corresponding genes are known, and are occasionally well-characterized, they do not have a specific known function. It is common to find this type of proteins in studies on microorganisms isolated from extreme environments because in many cases no studies have been carried out on their proteomes, or the isolated proteins have

not been characterized. It is worth mentioning that the new techniques used in genomics, such as NGS used in this study, have greatly facilitated the discovery of new proteins. They are fast methods that allow the identification of new genes and the amino acid sequence of hitherto unknown proteins. The problem is the identification of the function of these proteins since the techniques for this purpose (e.g. microarrays) are slower and more expensive. It cannot be discarded that these proteins belong to microeukaryotes that have not been identified in this study.

Once the functions of the proteins have been identified, the results obtained in each sample at 0ºC and 4ºC could be compared. These differences can be observed in the sector diagrams (Fig. 5 A, D).

When the diagram of M1 at 0ºC was compared with that of M1 at 4ºC (without considering the category of uncharacterized proteins), the most abundant category in both temperatures was "Protein synthesis". The second most abundant category present at 0ºC, "Transmission of genetic information and cell cycle", disappeared from the proteome when temperature increased (Fig. 5A). In addition, at 4ºC the second major category was "Adaptation to atypical conditions". It is worth mentioning the decrease of categories related to energy production (metabolism of macromolecules, Krebs cycle, electron transport chain, and ATP synthesis) that is observed when temperature increases. From these data, it can be deduced that the microorganisms present in the M1 sample do not withstand the increase in temperature.

In the case of sample M2, the major category was "Protein synthesis", followed by "Transmission of genetic information and cell cycle" in both conditions. In a similar way to what was observed in M1, when the temperature increased to 4ºC, the category size corresponding to "Adaptation to atypical conditions" increased while the categories related to obtaining energy decreased (especially those related to the Krebs cycle, electron transport chain, and ATP synthesis). Therefore, the conclusion is similar to the case of M1; although in this condition the microorganisms would adapt better, and a higher or more continuous increase in temperature would be necessary to cause the death of the microorganisms.

When the study of the proteomes of the M3 sample was carried out, no broad differences were observed for the relevant categories (Fig. 5C). The group "Adaptation to atypical conditions" decreased with increasing temperature (although the number of proteins was the same at 0ºC and 4ºC), while the categories related to "Transmission of genetic information and cell cycle" decreased. It could be deduced that to a lesser extent than in the previous cases, the correct development of the microorganisms in the M3 sample was altered by the increase in temperature.

Finally, when studying the distribution of the functional categories of proteins in the M4 sample, the group of "Uncharacterized proteins" was much more abundant at 4ºC than at 0ºC (Fig. 5D). It is noticeable that both diagrams were very similar, especially regarding the relevant categories to assess their adaptation. In this case, the adaptation to the increase in temperature was much better than in the other samples.

IV. Supplementary References

al-Bar OAM, O'Connor CD, Giles IG Akhtar M. 1992. d-alanine: d-alanine ligase of *Escherichia coli*. Expression, purification and inhibitory studies on the cloned enzyme. Biochemical Journal 282:747-752. doi: 10.1021/acs.jafc.0c00682.

Beassoni PR, Gallarato LA, Boetsch C, Garrido MN, Lisa AT. 2015. *Pseudomonas aeruginosa* Exopolyphosphatase Is Also a Polyphosphate: ADP Phosphotransferase. Enzyme Research 2015:1-13. https://doi.org/10.1155/2015/404607.

de Mello Serrano GC, e Silva Figueira TR, Kiyota E, Zanata N, Arruda P. 2012. Lysine degradation through the saccharopine pathway in bacteria: LKR and SDH in bacteria and its relationship to the plant and animal enzymes. FEBS Letters. 586:905-911. doi: 10.1016/j.febslet.2012.02.023.

Doherty GP, Meredith DH, Lewis PJ. 2006. Subcellular Partitioning of Transcription Factors in *Bacillus subtilis*. J Bacterio. 188: 4101-4110. doi: 10.1128/JB.01934-05.

Fukui T, Shiomi N, Doi Y. 1998. Expression and Characterization of (R)-Specific Enoyl Coenzyme A Hydratase Involved in Polyhydroxyalkanoate Biosynthesis by *Aeromonas caviae*. J Bacteriol 180:667-673. doi: https://doi.org/10.1128/JB.180.3.667-673.1998.

García-Descalzo L, García-López E, Alcázar A, Baquero F., Cid, C. 2014. Proteomic analysis of the adaptation to warming in the Antarctic bacteria *Shewanella frigidimarina.* Biochimica et biophysica acta. 1844:2229-2240. doi: 10.1016/j.bbapap.2014.08.006.

Hines JK, Chen X, Nix JC, Fromm HJ, Honzatko RB. 2007. Structures of Mammalian and Bacterial Fructose-1,6-bisphosphatase Reveal the Basis for Synergism in AMP/Fructose 2,6-Bisphosphate Inhibition. JBC. 282:36121-36131. doi: 10.1074/jbc.M611104200.

Jeong W, Cha MK, Kim IH. 2000. Thioredoxin-dependent Hydroperoxide Peroxidase Activity of Bacterioferritin Comigratory Protein (BCP) as a New Member of the Thiol-specific Antioxidant Protein (TSA)/Alkyl Hydroperoxide Peroxidase C (AhpC) Family. JBC. 275:2924-2930. doi: 10.1016/j.freeradbiomed.2013.07.036.

Johnson, J., Lackner, L., Hale, C., and de Boer, P. (2004). ZipA is required for targeting of D MinC/DicB, but Not D MinC/MinD, complexes to septal ring assemblies in Escherichia coli. J. Bacterio. 186, 2418–2429. doi: 10.1128/JB.186.8.2418-2429.2004

Joyce MA, Fraser ME, Brownie ER, James MNG, Bridger WA, Wolodko WT. 1999. Probing the Nucleotide-Binding Site of *Escherichia coli* Succinyl-CoA Synthetase†. Biochemistry. 38:7273-7283. doi: 10.1006/jmbi.1998.2325.

Mondini A, Donhauser J, Itcus C, Marin C, Perșoiu A, Lavin P, Frey B, Purcarea C. 2018. High-throughput sequencing of fungal communities across the perennial ice block of Scărișoara Ice Cave. Annals of Glaciology. 59:134-146. https://doi.org/10.1017/aog.2019.6.

Ramón-Maiques S, Marina A, Guinot A, Gil-Ortiz F, Uriarte M, Fita I, Rubio V. 2010. Substrate Binding and Catalysis in Carbamate Kinase Ascertained by Crystallographic and Site-Directed Mutagenesis Studies: Movements and Significance of a Unique Globular Subdomain of This Key Enzyme for Fermentative ATP Production in Bacteria. Journal of Molecular Biology. 397:1261-1275. https://doi.org/10.1016/j.jmb.2010.02.038.

TitzB, Rajagopala SV, Ester C, HäuserR, Uetz P. 2006. Novel Conserved Assembly Factor of the Bacterial Flagellum. J Bacteriol. 188:7700-7706. doi: 10.1128/JB.00820-06.

Zellmeier S, Schumann W, Wiegert T. 2006. Involvement of Clp protease activity in modulating the *Bacillus subtilis* σ^{W} stress response. Molecular microbiology. 61:1569-1582. https://doi.org/10.1111/j.1365-2958.2006.05323.x.
